# Relative Validity of Interviewer-Administered 24-Hour Recalls Collected By Telephone and In-person Compared With Weighed Food Records Among Rural Sri Lankan Adults

**DOI:** 10.1016/j.cdnut.2026.107672

**Published:** 2026-03-12

**Authors:** Caroline A Joyce, Christine P Stewart, Charles D Arnold, Hasara Sitisekara, Bess L Caswell, Sonja Y Hess, Amy Margolies, Thilanka Ranatunga, Thushanthi Perera, Deanna K Olney, Renuka Silva, Aulo Gelli

**Affiliations:** 1 University of California Davis, Institute for Global Nutrition and Department of Nutrition, Davis, CA, United States; 2 Wayamba University of Sri Lanka, Department of Nutrition and Dietetics, Makandura, Gonawila, Sri Lanka; 3 United States Department of Agriculture, Agricultural Research Service, Western Human Nutrition Research Center, Davis, CA, United States; 4 International Food Policy Research Institute, Nutrition, Diets, and Health Unit & Poverty, Gender, and Inclusion Unit, Washington, DC, United States

**Keywords:** 24-hour recall, dietary assessment, validation, cost analysis, weighed food records

## Abstract

**Background:**

Quantitative dietary assessment is hindered by cost, time, and logistical burdens. Collecting 24-hour recall (24HR) dietary data by phone may be particularly advantageous in rural areas.

**Objectives:**

To assess the criterion and relative validity and costs of phone compared with in-person 24HR versus observed weighed food records (WFR), a reference measure for true intake.

**Methods:**

We used a randomized crossover design to collect phone and in-person 24HRs from 103 rural Sri Lankan adults, each matched to a WFR collected on the reference day. Agreement was assessed using equivalence testing with 15% error bounds and concordance correlation coefficients. Memory and portion size estimation errors were examined using descriptive statistics. Activity-based costing was used to estimate 24HR costs using expenditures and micro-costing.

**Results:**

Compared with WFR, respondents underreported energy intake by 263 kcal (12.6%) in phone 24HR and 229 kcal (10.9%) in in-person 24HR, primarily due to portion size misestimation. Mean intakes of protein and 6 micronutrients were equivalent between phone 24HR and WFR. Although no nutrients were equivalent for in-person recalls, the two recall methods had relative equivalence for energy, carbohydrates, fat, zinc, and niacin. The mean concordance correlation coefficient was slightly higher for phone recalls, whereas memory error was lower for in-person recalls. Costs per respondent were $254 for in-person 24HR and $186 for phone 24HR in a most likely implementation scenario.

**Conclusions:**

Among rural Sri Lankan adults, phone-based 24HR were comparable to in-person 24HR in estimating nutrient intakes, although both methods underestimated true intakes. Phone-based recalls were less expensive and performed at least as accurately as those collected in-person.

## Introduction

Twenty-four-hour recalls (24HR) have been widely used to assess dietary intake since the 1960s [1]. Although interviews were traditionally conducted in-person and recorded using pen and paper, technological advancements in recent decades have enabled data to be collected and recorded electronically [2]. Nevertheless, collection and use of individual-level quantitative dietary intake data remains constrained by cost; time; logistical complexity; and technical capacity for data collection, analysis, interpretation [3,4]. The COVID-19 pandemic required rapid adaptations to research studies, spurring many dietary assessments to collect data by telephone. For example, an intervention evaluation in Sri Lanka, which was designed before the COVID-19 pandemic, needed to expedite data collection in anticipation of impending stay-at-home orders in the country. As a result, the study team shifted from in-person 24HR to the collection of 24HR by telephone. Despite expected challenges with respect to portion size estimation, it was thought that the phone-based method could be logistically easier to execute and would also likely be less costly than in-person interviews [5,6].

Only a few studies to date have compared the validity of both phone and in-person 24HR [[7], [8], [9], [10], [11], [12]]. Tran et al. [7] validated self-reported energy intake from phone and in-person recalls against energy expenditure data from doubly-labeled water and found that although both recall methods underestimated total energy, there was no difference in mean energy intake between the two recall methods [7]. Five other studies assessed phone and in-person 24HR, but only as a comparison between the methods, without gold standard measures of dietary intake (i.e., direct observation, weighed food record [WFR], or biomarkers) [[8], [9], [10], [11], [12]]. Two studies which validated phone-based 24HR against WFR found few differences between self-reported and observed mean energy or nutrient intakes; however, they did not include a similar comparison between in-person interviewer-administered 24HR and WFR [13,14].

All of the aforementioned studies were conducted among North American or Western European adults, which limits their generalizability [[7], [8], [9], [10], [11], [12], [13], [14]]. Given that over 94% of people in low- and middle-income countries have access to mobile phones, conducting 24HR by phone has become an increasingly feasible method of data collection [15]. Moreover, phone-based interviews eliminate the need to transport and potentially house enumerators during data collection and may be less burdensome for study participants, which could increase study response rates [16,17]. Despite the advantages from an implementation standpoint, it is unclear whether phone-based 24HRs provide data that are as accurate as in-person recalls. There are also important gaps regarding the costs of undertaking dietary assessment by phone in low- and middle-income countries, which are needed to inform decisions about data collection methods [18].

The objective of this study was to assess and compare the criterion validity and costs of phone-based 24HR relative to those collected in-person, using WFR as a reference measure for true intake. Our specific aims were to estimate mean nutrient intakes using the three data collection methods; to assess the validity of each 24HR method against WFR to measure mean energy, macronutrient, and micronutrient intakes; to compare the relative validity of the 24HR methods; to assess the extent and sources of error in phone and in-person recalls; and to assess the costs of each recall method. The findings from this study may provide evidence for the validity of an alternative and potentially less costly method of dietary assessment for future population-based studies.

## Methods

### Study design

The study used a randomized crossover design to compare the performance of phone and in-person interviewer-administered 24HR relative to WFR. All participants completed both recall modes, with the order of administration randomized such that half of the participants completed a WFR followed by the phone-based 24HR and, three to seven days later, a second WFR followed by the in-person 24HR. The other half completed the recalls in the opposite order. This design allowed for a within-person comparison of recall methods while controlling for participant-specific characteristics and dietary practices.

### Study population

We recruited adults 18 to 69 y old from four rural villages in Sri Lanka—two in Monaragala District and two in Mullaitivu District. Volunteers from the target villages gathered at a central location for enrollment on a pre-specified day. Individuals were eligible to participate if they lived in one of the study villages and were within the target age range at the time of enrollment. They were not eligible if they did not provide consent or had significant cognitive impairment. Participants were enrolled until the required sample size and an equal number of males and females in each district had been recruited.

**FIGURE 1:**
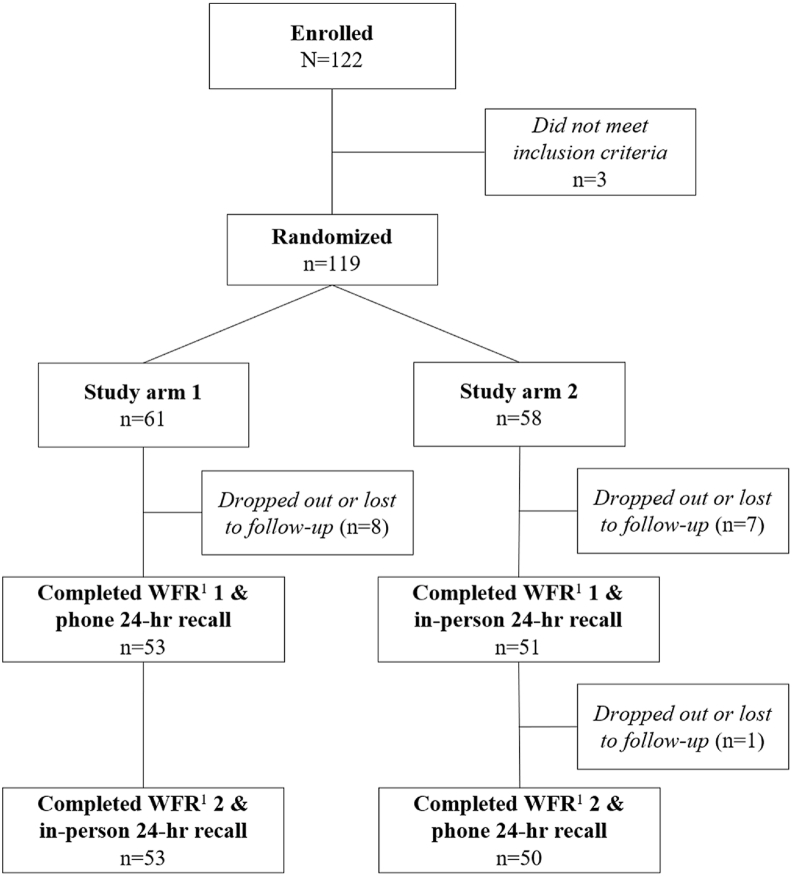
CONSORT flow diagram of participants through a phone and in-person 24-hour recall validation study. WFR, weighed food record.

Our sample size was selected assuming an equivalence margin within ±15% of total energy intake, 80% power, and α of 0.05. Considering a mean intake of 1902 kcal and an SD of 692.6 kcal in a recent dietary intake assessment in rural Sri Lanka, a minimum of 101 respondents were required [5,19]. After accounting for a potential 10% loss to follow-up, the target sample size was 112 participants. Ultimately, 122 participants were enrolled, since 19 participants dropped out (*n* = 15) or were removed from the study early due to concerns around the safety of the enumerators (*n* = 4). Safety concerns were related to unpredictable behavior of one or more household members, which could have been due to alcohol use or mental health issues (Figure 1). In all cases, enumerators concluded the visit, and the respondent was replaced in the study. Participants were randomized to the two study arms (phone or in-person 24HR first) using random number generation in Stata version 17 [20]. Participant randomization was stratified by district.

### Data collection

Local nutrition graduate students, including native Tamil and Sinhala speakers, were recruited and paid to undertake data collection. All enumerators participated in five days of training at the Wayamba University of Sri Lanka, followed by four days of piloting and debriefing in the community. Data were collected from February to March 2024. Sociodemographic information was collected during the enrollment visit. Dietary intake was assessed using three methods: phone-based 24HR, in-person 24HR, and WFR.

On day 1 of the study, participants completed a WFR. During the first WFR, the enumerators also collected the participants’ height and weight using portable stadiometers (Seca 213) and digital scales (Seca 813). On day 2, enumerators administered the first 24HR method that the individual had been randomized to provide. Three to seven days later, each participant completed a second WFR, and the other form of 24HR the following day (Figure 1). Although recollection of the 24HR procedure may decrease over time, a longer washout period was not feasible in this study; therefore, recall order was randomized to balance potential learning effects across methods. Both recall periods were included in the analysis, and comparisons between recall methods were conducted within individuals, with each 24HR compared to its corresponding WFR.

Although the enumerators recorded use of supplements during WFR data collection, the supplement data were not incorporated into the analysis. Only 3% of respondents used a vitamin or mineral supplement, and the quantity and frequency data were often missing. All data were recorded in electronic SurveyCTO (Dobility, Inc) forms developed by the study team using Galaxy Tab A tablets (Samsung SM-X205NZAA) [21,22].

#### WFRs

To collect the WFRs, enumerators shadowed participants from approximately 06:30 until the last meal of the day (18:15–20:30). Using digital scales accurate to one gram (Camry Industries Company Ltd) [23], enumerators recorded the weights of all foods and beverages consumed during the hours they were present, including uneaten portions, which were subtracted during data processing. Whenever possible, the enumerators documented recipe data, including ingredients and gram weights, the cooking method, and the final cooked weight. If a recipe was recorded but the details could not be captured, enumerators recorded the main ingredients. All meals consumed by the respondents were consumed on individual plates, rather than from shared plates among household members.

#### Multiple-pass 24HR recall

Twenty-four-hour recalls were conducted using the multiple-pass method [24]. The food list available in the tablet-assisted personal interview form included nearly 800 individual food items tailored to the study population. In the first pass, respondents were asked to list all foods and beverages consumed the previous day, from waking up until bedtime. For each item, the enumerators asked whether the food or beverage was consumed while the WFR enumerator was present. During the second pass, enumerators solicited detailed descriptions of each food or beverage from pass 1, including the time of consumption and whether any condiments were added. In pass 3, respondents estimated the volume of foods/beverages, condiments, and leftovers, as applicable. During in-person interviews, enumerators asked respondents to report portion sizes using 3-dimensional household utensils and dishware (e.g., tablespoons, coconut spoons, and drinking glasses), visual aids (e.g., 2-dimensional roti), common sizes of local foods, and descriptions (e.g., “palm-sized pieces”) from a pre-specified list. In the fourth pass, enumerators reviewed all foods and beverages with respondents to ensure that there were no errors or forgotten foods.

The phone 24HRs were modified to reduce interview length and enable portion size estimation without visual aids. We consolidated the second and third passes into a single pass and removed some questions (e.g., brand names of packaged foods and certain portion size descriptors). Although each reported food or beverage corresponded to a more limited set of portion size units, the phone recall used the same household utensils and dishware, common sizes of local foods, and “palm-size” descriptions as in the in-person recalls. The volume of each participant’s reported units was based on their own estimation and/or verbal descriptions from the enumerator.

#### Costs

Financial and economic cost data were collected from January to September 2024. Financial costs were estimated using Wayamba University’s expenditure records. University of California, Davis (UC Davis) staff recorded the time they spent on assessment activities weekly throughout the project.

### Data analysis

A statistical analysis plan was developed and posted publicly prior to analysis (https://osf.io/57tgf). We used Stata version 17 for data cleaning, management, and descriptive analyses [20]. We generated descriptive statistics to summarize the demographic characteristics of the sample. All dietary data processing and nutritional analyses were conducted by a single analyst to ensure consistency across methods, with regular consultation from senior coauthors and input from collaborators at the Wayamba University to support accurate interpretation of local foods and recipes. Cost analyses were conducted separately by a researcher at the International Food Policy Research Institute with input from team members from the Wayamba University and UC Davis.

To estimate the quantities of foods reported in the 24HR, we applied gram weight conversion factors corresponding to each food type and portion unit. The conversion factors were estimated from triplicate laboratory weighing and analyses of common Sri Lankan recipes cooked by the study team and the USDA FoodData Central [25]. After accounting for leftovers, three food-level observations in the WFR yielded implausible negative amounts due to unresolved discrepancies in container or leftover weights and were therefore excluded from the analysis.

When recipes or packaged foods were reported in the 24HR, or when recipe data were not collected in the WFRs, we estimated nutrient compositions using standard recipes and a recipe calculator previously developed by the study team and publicly available on Open Science Framework (https://doi.org/10.17605/OSF.IO/4JKYR). The calculator incorporates yield factors (to account for water gains or losses), nutrient retention factors for cooking from the USDA, and ingredient-level nutrient data from the Sri Lankan food composition table [26,27]. For all data collection methods, we primarily used Sri Lankan food composition data to estimate nutrient values of ingredients and foods “as consumed” [26]. We supplemented food composition data with values from neighboring countries and the USDA when necessary [25]. Data from other countries’ food composition tables comprised ∼5% of the total food and nutrient composition databases used to estimate nutrient intakes. During analysis, we trimmed the 24HR data to the period during which the WFR enumerator was present to allow for direct comparison.

#### Mean nutrient intakes

After summing the total nutrients reported/consumed by each participant, we performed descriptive analyses to estimate the mean, SD, median, and IQR of intake for energy (kcal), macronutrients (carbohydrates [grams], fat [grams], and protein [grams]), and 11 micronutrients (iron [milligrams], calcium [milligrams], zinc [milligrams], thiamine [milligrams], niacin [milligrams], riboflavin [milligrams], folate [micrograms], vitamin A [micrograms of retinol equivalents; RE], vitamin C [milligrams], vitamin B6 [milligrams], and vitamin B-12 [micrograms]) by data collection method. We assessed histograms and scatter plots of the distribution of intakes. Any reported intake that was >3500 kcal or <800 kcal was flagged and individually reviewed for data entry errors and plausibility. After consulting with the enumerator who collected the data in question, all observations were retained.

#### Extent of agreement between data collection methods

Linear regression models were used to estimate the mean differences in log-transformed nutrient intakes between each 24HR method and the corresponding WFR. For each nutrient, the dependent variable was the log-transformed ratio of nutrient intake estimated by the 24HR to intake measured by the WFR for the same individual on the reference day, and the primary independent variable was the recall method (phone or in-person). Interviewer identification number and respondent sex were included as fixed effects in all models. Nutrient intakes were log-transformed because intake distributions are generally right-skewed.

We performed equivalence testing using the two one-sided test procedure, for which the resulting confidence intervals (CIs) are 90%, each with α = 0.05 [28]. Since the mean difference for log-transformed data is equivalent to the ratio of reported:actual nutrient intake, the 15% bound is equivalent to a ratio of observed to true intake between 0.85 and 1.15 [29,30]. The 24HR methods were considered equivalent to the WFR if the 90% CI around the mean difference fell within ±15% of the WFR value for each nutrient [[29], [30], [31], [32], [33]]. The same procedure was used to test whether the differences in nutrient intake estimated by phone 24HR and WFR were equivalent to the differences between in-person 24HR and WFR.

We also assessed the extent of agreement between the recall methods and WFR using concordance correlation coefficients (CCCs) and Bland-Altman plots [34,35]. The Bland-Altman plots illustrate the mean differences between reported and actual nutrient intake for each method and the limits of agreement, which correspond with the range in which 95% of the differences between methods occur [34].

#### Sources of error

Memory error was estimated by the number of omissions (foods recorded in the WFR but not in the 24HR) and intrusions (foods reported in the 24HR but not in the WFR). Observations were matched using an iterative process. After merging by specific food code, we used three levels of categorization to align near-matches. For example, if “sprats, tempered, boiled with coconut milk” was recorded in the WFR, and “sprats, boiled with coconut milk” was recorded in the 24HR, we considered these foods a match. We individually reviewed the data after each round of matching. If a participant reported three instances of a given food in their 24HR but four instances were recorded in the WFR, the remaining instance in the WFR was categorized as an omission, and vice versa.

Portion size estimation error was assessed as the difference between the mean observed and reported intakes of each food or beverage, and as a proportion of true intake ((WFR mean − 24HR mean)/WFR mean). We analyzed the extent of error in grams and kilocalories for any food with at least two observations in all three data collection methods, and we reported foods that contributed the greatest proportion of the total underestimation error in each recall method.

#### Costs

We estimated the incremental financial and economic costs of undertaking phone and in-person 24HR using societal and payer perspectives, i.e., we identified and included the costs of all resources needed to conduct the survey [36,37]. We used a mixed-methods approach to assess and allocate resource use to activity and input costs, using financial expenditure data combined with micro-costing. We allocated expenditures by activities using the ingredients approach method that estimates costs for all resources used, including volunteer time and donated supplies [18].

Financial costs were calculated from actual expenditures associated with administering each 24HR method. Because the validation study employed a crossover design, using the same enumerator team to collect both 24HRs and WFRs, the per-design cost analysis included enumerator transportation, accommodation, and meal costs for both in-person and phone-based interviews.

Economic costs captured the opportunity cost of participants’ time, which were estimated using the value of incentives provided (based on local daily wage rates). Researcher staff time at UC Davis was recorded via weekly time diaries. Start-up activities included adaptation and programming of the data collection forms, development of training materials, and obtaining ethical approvals, while recurrent activities included dietary interview implementation and associated travel, accommodation, and meal costs for enumerators under the per-design conditions.

To reflect more typical field implementation, we presented two additional costing scenarios (phone survey scenarios 1 and 2) (Supplemental Table 1). Both excluded enumerator travel and meal costs, assuming the interviews would be conducted from a central duty station. Scenario 1 (the most likely implementation scenario) applied interview-time allocations estimated by the principal investigator, whereas scenario 2 used the actual interview-time distributions observed in the study for sensitivity analysis.

Costs related to WFR data collection, external research activities, and reference data preparation (e.g., food lists, portion size conversions, recipe data, and food composition tables) were excluded, as these were largely developed in prior studies and were not expected to differ by data collection method. All expenditures were coded under standardized cost categories [38], converted to 2024 USD, and annualized using a 3% discount rate (2-y lifespan for weight scales and props; 4-y lifespan for tablets) [39].

### Ethical considerations

The study protocol was reviewed and approved by the Wayamba University of Sri Lanka’s Ethics Review Committee and the International Food Policy Research Institute’s Institutional Review Board (IRB). The study protocol was also reviewed by the UC Davis IRB, which determined that it involved human subjects research but that the research team members from UC Davis were “not engaged” in human subjects’ research as defined by federal regulations (45 CFR 46). Therefore, UC Davis IRB approval was not required. During the enrollment visit, enumerators read the informed consent form to participants in their native language (Sinhala or Tamil). Written informed consent was obtained from all subjects and recorded in SurveyCTO.

## Results

The final study sample included 103 adults (Figure 1), 51% of whom were female (*n* = 53) (Table 1). The median age was 46 years, ranging from 21 to 68, and the median BMI was 22.8. Two-thirds of participants had at least a secondary school education (64%). Nearly half of the participants engaged in agriculture, including farming, fishing, or livestock (47%), and another quarter reported labor work as their primary occupation. On average, enumerators spent 12.8 h/d in each participant’s home collecting WFRs. Phone 24HR took 22 ± 8 min, and in-person 24HR took 24 ± 8 min.

**TABLE 1:** Baseline characteristics of participants in the phone 24-h recall (24HR) evaluation study among rural Sri Lankan adults (*n* = 103).

	Study arm 1, *n* (%) Phone 24HR first *n* = 53	Study arm 2, *n* (%) In-person 24HR first *n* = 50
Sex (female)	25 (47.2)	28 (65.0)
Age, (y, mean ± SD)	47.9±11.5	45.1±12.6
BMI, (mean ± SD)	23.2±4.7	23.1±4.3
Household size, (mean ± SD)	3.7±1.3	3.5±1.2
Head of household (yes)	39 (73.6)	31 (63.3)
Education		
No schooling	1 (1.89)	1 (2.0)
Primary	21 (39.6)	14 (28.0)
Secondary	28 (52.8)	30 (60.0)
Advanced (grade 12+)	3 (5.7)	5 (10.0)
Marital status		
Single	1 (1.9)	2 (4.0)
Married	41 (77.4)	39 (78.0)
Divorced/separated	5 (9.4)	4 (8.0)
Widowed	6 (11.3)	5 (10.0)
Occupation		
Agriculture (farming, livestock rearing, and/or fishing)	30 (56.6)	18 (36.0)
Homemaker	2 (3.8)	3 (6.0)
Labor work	11 (20.8)	15 (30.0)
Other	6 (11.3)	6 (12.0)
None	4 (7.6)	8 (16.0)
Primary source of household income		
Agriculture (farming, livestock rearing, and/or fishing)	34 (64.2)	17 (34.0)
Labor work	13 (24.5)	23 (46.0)
Other	6 (11.3)	10 (20.0)

### Mean nutrient intakes

The mean ± SD energy intake across all WFRs was 2098 ± 692 kcal. On average, 60% of energy came from carbohydrates, 29% from fat, and 11% from protein. In both recall methods, the mean reported intake for energy and most nutrients was lower than the corresponding WFRs (Table 2). Respondents underreported energy by 263 ± 623 kcal (12.6%) in phone recalls and by 229 ± 676 kcal (10.9%) in in-person recalls.

**TABLE 2:** Mean and standard deviation, median, and interquartile range of nutrient intakes by data collection method in the phone 24HR evaluation study among rural Sri Lankan adults (*n* = 103).

	Phone 24HR vs. WFR				In-person 24HR vs. WFR			
	Phone 24HR		Reference WFR		In-person 24HR		Reference WFR	
	Mean ± SD	Median (IQR)	Mean ± SD	Median (IQR)	Mean ± SD	Median (IQR)	Mean ± SD	Median (IQR)
Energy (kcal)	1833 ± 686	1685 (1357, 2135)	2095 ± 729	1870 (1569, 2584)	1872 ± 795	1642 (1319, 2181)	2100 ± 655	1924 (1664, 2606)
Carbohydrates (g)	273 ± 99	251(210, 320)	311 ± 109	286 (237, 383)	295 ± 132	260 (202, 347)	319 ± 97	298 (245, 384)
Fat (g)	60 ± 31	53 (36, 79)	69 ± 31	61 (49, 89)	56 ± 28	53 (37, 72)	67 ± 29	62 (47, 82)
Protein (g)	52 ± 23	47 (37, 64)	56 ± 22	49 (39, 72)	48 ± 21	41 (34, 62)	55 ± 18	51 (42, 67)
Calcium (mg)	324 ± 240	273.5 (152.9, 430.3)	307 ± 181	268.1 (163.8, 394.2)	275 ± 203	231.2 (119.3, 366.6)	318 ± 217	252.4 (180.5, 420.0)
Iron (mg)	10.7 ± 5.4	9.1 (7.4, 13.2)	11.5 ± 5.2	10.3 (7.4, 14.6)	9.9 ± 5.0	8.8 (5.9, 13.2)	11.6 ± 5.2	10.5 (7.8, 14.4)
Zinc (mg)	8.1 ± 3.2	7.3 (6.1, 9.9)	8.8 ± 3.2	8.2 (6.4, 11.3)	8.1 ± 3.7	6.8 (5.6, 9.7)	9.0 ± 3.0	8.3 (7.0, 10.8)
Vitamin A RE (μg)	298 ± 577	109.9 (47.7, 290.5)	315 ± 552	115.5 (57.3, 304.0)	188 ± 265	81.0 (36.0, 205.9)	252 ± 356	105.7 (58.5, 310.1)
Thiamine (mg)	0.5 ± 0.3	0.4 (0.3, 0.6)	0.5 ± 0.2	0.5 (0.3, 0.6)	0.4 ± 0.3	0.4 (0.3, 0.6)	0.5 ± 0.2	0.5 (0.3, 0.6)
Riboflavin (mg)	0.5 ± 0.3	0.4 (0.3, 0.7)	0.5 ± 0.2	0.5 (0.3, 0.7)	0.5 ± 0.3	0.4 (0.3, 0.6)	0.5 ± 0.2	0.5 (0.3, 0.6)
Niacin (mg)	8.9 ± 4.3	8.0 (6.0, 11.2)	9.7 ± 4.2	8.6 (6.8, 11.9)	8.8 ± 4.6	7.7 (5.4, 10.8)	9.9 ± 4.3	8.7 (6.9, 12.0)
Vitamin B-6 (mg)	1.1 ± 1.5	0.8 (0.6, 1.1)	1.1 ± 0.9	0.9 (0.6, 1.2)	1.1 ± 1.3	0.7 (0.5, 1.2)	1.0 ± 0.4	0.9 (0.6, 1.2)
Folate (μg)	159 ± 84	141.9 (97.5, 197.3)	162 ± 71	155.0 (101.8, 208.0)	140 ± 85	116.7 (75.9, 191.0)	161 ± 81	141.2 (100.2, 201.5)
Vitamin B-12 (μg)	1.0 ± 1.2	0.5 (0.2, 1.4)	0.8 ± 0.9	0.6 (0.2, 1.1)	0.7 ± 1.0	0.5 (0.2, 0.8)	0.8 ± 0.9	0.6 (0.1, 1.0)
Vitamin C (mg)	58 ± 75	36.4 (18.7, 58.7)	50 ± 40	35.2 (20.3, 66.0)	74 ± 205	25.3 (13.2, 62.9)	56 ± 56	39.5 (21.2, 73.4)

Abbreviations: 24HR, 24-hour recall; RE, retinol equivalents; WFR, weighed food record.

### Agreement between methods

**FIGURE 2:**
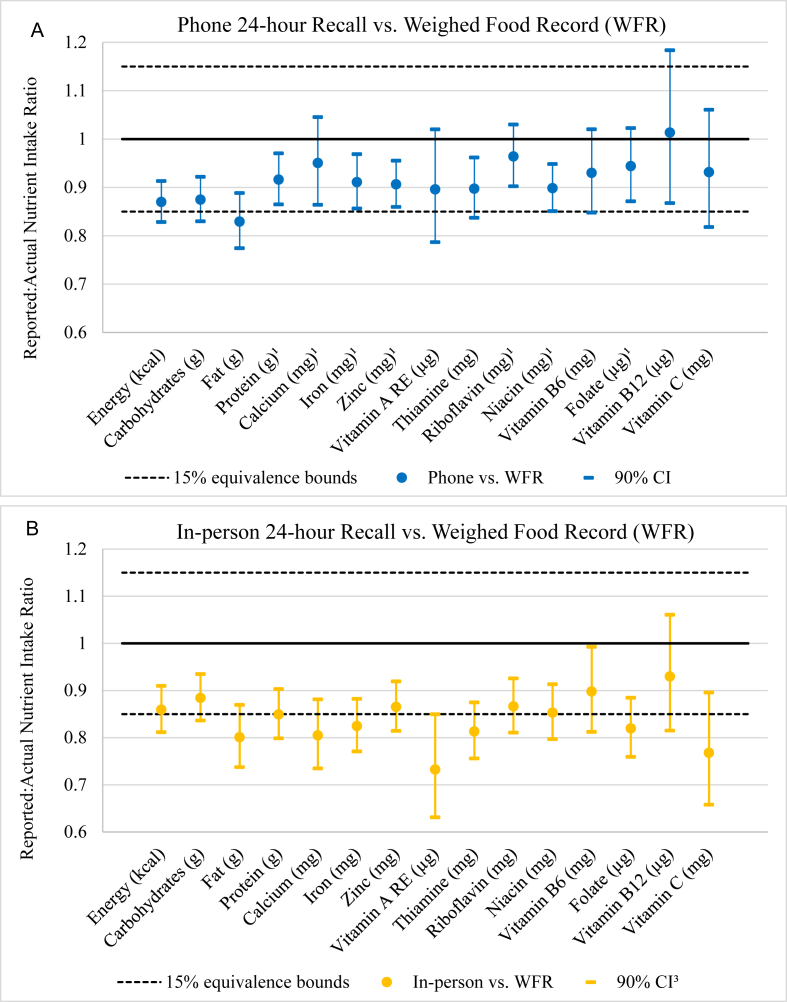
Equivalence testing of reported:actual nutrient intake ratios between (A) phone and (B) in-person 24-hour recalls and their corresponding weighed food records in the phone 24-hour recall evaluation study among rural Sri Lankan adults (*n* = 103). ^1^24-hour recall was equivalent to weighed food record for this nutrient. Linear regression models included interviewer and sex as fixed effects. CI, confidence interval.

**FIGURE 3:**
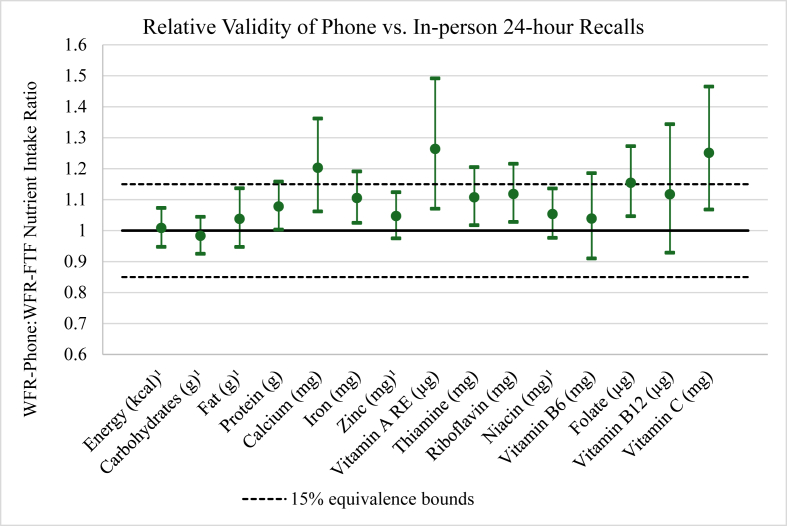
Equivalence testing of the differences between phone 24HR and WFR nutrient intake estimates relative to the differences between in-person 24HR and WFR in the phone 24HR evaluation study among rural Sri Lankan adults (*n* = 103). ^1^24-hour recall was equivalent to WFR for this nutrient. Linear regression models included interviewer and sex as fixed effects. 24HR, 24-hour recall; CI, confidence interval; WFR, weighed food record.

For the phone 24HR, protein, calcium, iron, zinc, riboflavin, niacin, and folate were equivalent to WFRs within a 15% margin. The mean ratios of micronutrient intakes estimated by phone-based 24HR relative to those measured by WFR ranged from 0.90 (90% CI: 0.79, 1.02) for vitamin A to 1.01 (90% CI: 0.87, 1.18) for vitamin B-12 (Figure 2 and Supplemental Table 2). In the in-person recalls, no nutrients were equivalent to WFR within 15%. Across both recall methods, the ratio of observed to recalled intake was <1.0 for all nutrients except vitamin B-12 in phone recalls. Vitamin A estimates from in-person recalls showed the greatest extent of underreporting [0.73 (90% CI: 0.63, 0.85)]. The differences between WFR and phone recall intake estimates and the differences between WFR and in-person recall intake estimates were equivalent for energy, carbohydrates, fat, zinc, and niacin (Figure 3 and Supplemental Table 2).

**FIGURE 4:**
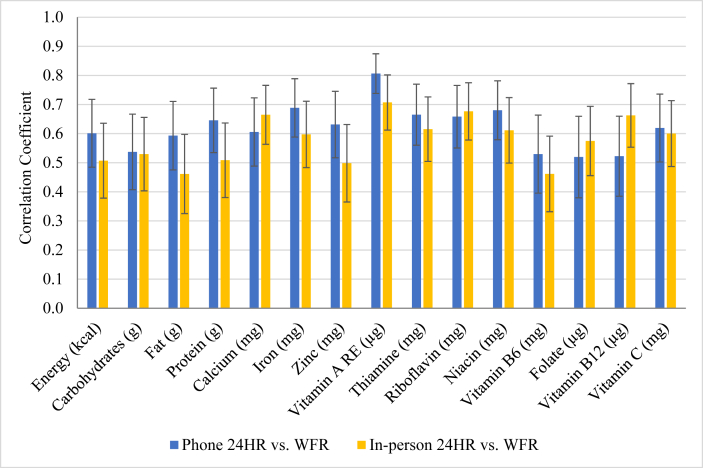
Concordance correlation coefficients comparing nutrient intakes reported in phone and in-person 24-hour recalls to their corresponding weighed food records in the phone 24-hour recall evaluation study among rural Sri Lankan adults (*n* = 103). Error bars express 95% CI.

The CCCs comparing 24HR to their corresponding WFRs were lowest for folate and vitamin B-12 in phone recalls (0.52 for both nutrients) and for vitamin B-6 and fat in in-person recalls (0.46 for both nutrients). The CCCs were highest for vitamin A (0.81 for phone recalls and 0.71 for in-person) (Figure 4 and Supplemental Table 3). The CCCs between phone recalls and WFR were higher than those between in-person recalls and WFR for all nutrients except calcium, riboflavin, folate, and vitamin B-12. The average CCC was 0.62 and 0.58 for phone and in-person recalls, respectively. In the Bland-Altman plots, the mean difference between phone recalls and WFRs was closer to zero for fat, protein, and all micronutrients except vitamin B-12, suggesting less bias than in-person recalls (Supplemental Figure 1). However, the distributions of differences did not follow a consistent pattern.

### Sources of error

Of the 1451 foods reported in phone-based recalls, 8% were not recorded in the preceding day’s WFR (*n* = 120), and 10% of WFR foods were omitted from the subsequent day’s phone recall (*n* = 145 of 1476; Table 3). Sugar, brewed black tea, milk powder, and coconut milk sauce were the most common omissions (*n* = 14, 13, 6, and 5 respondents, respectively). The same four items accounted for the most intrusions (*n* = 20, 14,10, and 6, respectively). Three quarters of respondents (*n* = 77) had at least one omission and/or intrusion in phone 24HR.

**TABLE 3:** Proportion of omissions and intrusions in phone and in-person 24HR compared with WFR, and the proportion of respondents who omitted or intruded food items in the phone 24HR evaluation study among rural Sri Lankan adults (*n* = 103).

	Phone 24HR vs. WFR		In-person 24HR vs. WFR	
	Frequency	Average kcal per omission/intrusion	Frequency	Average kcal per omission/intrusion
		mean ± SD		mean ± SD
Total foods observed	1476	—	1473	—
Total foods recalled	1451	—	1480	—
Errors of intrusion (% of observed foods)	120 (8.1)	73 ± 107	105 (7.1)	74 ± 107
Errors of omission (% of observed foods)	145 (9.8)	80 ± 128	98 (6.7)	68 ± 78
Recall days				
Recalls with intrusion errors (% of respondents)	54 (52.4)	—	47 (45.6)	—
Recalls with *n* intrusions (% of respondents)				
1 intrusion	19 (35.2)	102 ± 119	20 (42.6)	84 ± 91
2 intrusions	19 (35.2)	74 ± 130	10 (21.3)	92 ± 177
3 intrusions	9 (16.7)	77 ± 116	9 (19.1)	64 ± 74
4 intrusions	2 (3.7)	35 ± 31	5 (10.6)	54 ± 53
≥5 intrusions	5 (9.3)	59 ± 56	3 (6.4)	78 ± 115
Recalls with omission errors (% of respondents)	63 (61.2)	—	47 (45.6)	—
Recalls with *n* omissions (% of respondents)				
1 omission	27 (42.9)	96 ± 139	19 (40.4)	92 ± 102
2 omissions	14 (22.2)	89 ± 154	11 (23.4)	78 ± 87
3 omissions	11 (17.5)	51 ± 64	12 (25.5)	67 ± 72
4 omissions	4 (6.4)	154 ± 233	4 (8.5)	45 ± 45
≥5 omissions	7 (11.1)	58 ± 59	1 (2.1)	16 ± 19

Abbreviations: 24HR, 24-hour recall.; WFR, weighed food record.

In in-person recalls, 7% of reported foods were not present in the previous day’s WFR (*n* = 105/1020). Of the 1473 foods recorded in the WFRs, 98 were omitted from the subsequent day’s recall (7%). As in the phone survey, sugar was the most omitted food (13 respondents), followed by coconut milk sauce, brewed black tea, and milk powder (*n* = 11, 9, and 7 respondents, respectively). Sugar, coconut milk sauce, and brewed black tea were also the most common intrusions (*n* = 12, 6, and 6 respondents, respectively), followed by papadum, a type of lentil-based chip (*n* = 5). Just over 60% of respondents (*n* = 63) had at least one omission and/or intrusion in in-person recalls.

Polished medium-grain red rice, split red lentils with coconut milk, and coconut roti accounted for the largest proportion of the total underestimation error in phone recalls (Table 4). On average, participants underreported their energy intake from red rice by −57 kcal (SD: −87, −27), red lentils by −57 kcal (SD: −96, −18), and coconut roti by −297 kcal (SD: −396, −198). In the in-person 24HR, parboiled medium-grain red rice, coconut roti, and sugar accounted for the largest proportion of energy underreporting. On average, participants underreported their energy intake from red rice by −152 kcal (SD: −271, −34), coconut roti by −436 kcal (SD: −988, 115); and sugar by −11 kcal (SD: −17, −4).

**TABLE 4:** Mean portions reported in phone and in-person 24HR compared with their corresponding WFR in the phone 24HR evaluation study among rural Sri Lankan adults (*n* = 103 participants).

Food	Phone 24HR vs. WFR				In-person 24HR vs. WFR			
	Grams	*P* value	Kcal	*P* value	Grams	*P* value	Kcal	*P* value
Polished medium-grain red rice	*n* = 183 food observations				*n* = 186			

Observed, mean ± SD	292 ± 116	<0.01	399 ± 158	<0.01	287 (107)	0.89	392 ± 146	0.88
Recalled, mean ± SD	250 ± 99		341 ± 135		285 (145)		390 ± 198	
Difference, mean (95% CI)	−42 (−64, −20)		−57 (−87, −27)		−2 (−28, 24)		−3 (−38, 33)	
% error, mean (95% CI)	−14 (−22, −7)		−14 (−22, −7)		−1 (−10, 8)		−1 (−10, 8)	

Split red lentils with coconut milk	*n* = 57				*n* = 47			

Observed, mean ± SD	91 ± 55	0.02	190 ± 126	<0.01	78 (41)	0.05	147 ± 80	0.06
Recalled, mean ± SD	70 ± 40		132 ± 77		61 (42)		116 ± 79	
Difference, mean (95% CI)	−21 (−39, −3)		−57 (−96, −18)		−17 (−34, −0)		−31 (−63, 1)	
% error, mean (95% CI)	−23 (−43, −4)		−30 (−51, −10)		−22 (−43, −1)		−21 (−43, 1)	

Coconut roti	*n* = 10				*n* = 6			

Observed, mean ± SD	141 ± 43	<0.01	431 ± 137	<0.01	134 (61)	0.04	595 ± 549	0.16
Recalled, mean ± SD	47 ± 15		134 ± 44		55 (21)		158 ± 60	
Difference, mean (95% CI)	−94 (−126, −63)		−297 (−396, −198)		−79 (−142, −15)		−436 (−988, 115)	
% error, mean (95% CI)	−67 (−89, −44)		−69 (−92, −46)		−59 (−106, −11)		−73 (−166, 19)	

Parboiled medium-grain red rice	*n* = 13				*n* = 9			

Observed, mean ± SD	336 ± 96	0.02	394 ± 113	0.02	409 (96)	0.02	480 ± 113	0.02
Recalled, mean ± SD	234 ± 57		274 ± 66		280 (132)		328 ± 155	
Difference, mean (95% CI)	−102 (−181, −24)		−120 (−212, −28)		−130 (−231, −29)		−152 (−271, −34)	
% error, mean (95% CI)	−30 (−54, −7)		−30 (−54, −7)		−32 (−56, −7)		−32 (−56, −7)	

Sugar	*n* = 150				*n* = 139			

Observed, mean ± SD	14 ± 9	0.60	54 ± 35	0.60	14 (9)	<0.01	55 ± 34	<0.01
Recalled, mean ± SD	14 ± 7		52 ± 27		11 (6)		44 ± 23	
Difference, mean (95% CI)	−0 (−2, 1)		−2 (−9, 5)		−3 (−4, −1)		−11 (−17, −4)	
% error, mean (95% CI)	−3 (−16, 9)		−3 (−16, 9)		−19 (−31, −7)		−19 (−31, −7)	

Foods listed are those which were observed/reported by at least 2 participants per data collection method, and which contributed the greatest proportion of portion size estimation error in each recall method.

Abbreviations: 24HR, 24-hour recall; CI, confidence interval; WFR, weighed food record.

### Costs

The per-design cost of undertaking dietary assessment was $254.08 per respondent for in-person 24HR and $245.74 per respondent for phone 24HR, which included enumerator transportation and accommodation in the field during data collection. Analyses reflecting the likely implementation scenario for phone surveys (i.e., excluding field costs) found unit costs of $185.17 to $185.97, resulting in cost savings of $68.91 to $68.11 (27%) per survey (Table 5 and Supplemental Figure 2). When comparing the in-person survey costs compared with phone-based survey scenarios, fieldwork accounted for the greatest difference in overall survey costs. Nearly 33% of the total cost of the in-person survey was dedicated to data collection, which included enumerator salaries, transportation, meals, and accommodation. In contrast, data collection costs accounted for <15% of the total cost of the phone-based survey (assuming phone survey scenarios 1 or 2), which was primarily driven by enumerator salary costs.

**TABLE 5:** Total and unit costs (USD) for dietary assessment using phone-based or in-person 24-h recall in the phone 24-h recall evaluation study among rural Sri Lankan adults.

Primary activity	Per-design expenditures^1^				Phone survey scenario 1^1^		Phone survey scenario 2^1^	
	In-person ($)	% of total	Phone ($)	% of total	Phone ($)	% of total	Phone ($)	% of total
Administration	333.73	1.3	333.73	1.3	333.73	1.7	333.73	1.7
Planning / microplanning	1829.28	6.9	1634.10	6.5	1634.10	8.5	1634.10	8.6
Materials development	1512.85	5.7	1469.48	5.8	1469.48	7.7	1469.48	7.7
Procurement^2^	946.91	3.6	918.71	3.6	918.71	4.8	918.71	4.8
Training^2^	4181.60	15.8	4018.96	15.9	3812.32	19.9	3793.76	19.9
Survey preparation^2^	963.18	3.6	898.12	3.5	773.88	4.0	771.81	4.0
Data collection^2^	8639.70	32.7	8639.70	34.1	2813.96	14.7	2752.09	14.4
Data management and analysis^4^	8016.66	30.3	7398.60	29.2	7398.60	38.6	7398.60	38.8
Total	26,423.91	—	25,311.41	—	19,154.79	—	19,072.29	—
Number of respondents (*n*)	104^3^	—	103	—	103	—	103	—
Cost per interview	254.08	—	245.74	—	185.97	—	185.17	—

1 Per design expenditures included all costs incurred to conduct the dietary assessment study, excluding those associated with external research and preparation of the reference data (e.g., food lists, portion size conversions, recipe data). Relative to per-design expenditures, phone survey scenario 1 (the likely implementation scenario) excludes the cost of enumerators’ meals, transportation, and accommodation in the field, and it applies an allocation of interview times based on estimates from the principal investigator. Phone survey scenario 2 also excludes the cost of enumerators’ meals, transportation, and accommodation in the field but applied the actual allocation of interview times based on survey findings as in the per-design cost analysis.

2 Procurement included equipment and incentives for respondents and field officers. Training costs included salaries and accommodation at the University during enumerator training. Survey preparation and data collection included all staff salaries, transportation, and accommodation in the field during pilot testing and the data collection period (as applicable), respectively.

3 One additional participant completed an in-person interview but dropped out of the study before completing the phone interview

4 Data management and analysis included preparation of respondent-level nutrient intake datasets by recall method. These costs reflect study data processing only and exclude any external or validation analyses.

## Discussion

Our study sought to determine the validity of telephone-based 24HR relative to those conducted in-person, using WFRs to estimate true intake, and to compare the costs of the two 24HR approaches. In this population of rural Sri Lankan adults, energy intake was underreported by 12.6% in the phone recalls and by 10.9% in the in-person recalls. The degree of underreporting by macronutrient was similar, and the magnitude of error in energy, carbohydrate, fat, zinc, and niacin intake estimates were equivalent between the recall methods. The extent of memory error was low in both phone and in-person 24HR (6%–10% of observations omitted or intruded). Costs per respondent were $254 for in-person 24HR and $186 for phone 24HR unit costs under a likely implementation scenario, resulting in a cost savings of about $68 per survey for the phone-based survey.

Our finding of energy underreporting is consistent with previous studies. A review of dietary assessment methods found that adults underreported energy intake by 8% to 30% in 24HR compared to energy expenditure estimated by doubly-labeled water [40]. Tran et al. [7] used doubly-labeled water to validate both phone and in-person 24HR and found that relative to total energy expenditure, the mean reported energy intake was −391 kcal (−15%) in phone surveys and −471 kcal (−18%) in in-person surveys, but that the difference between the recall methods was not statistically significant [7]. Four additional studies found no significant differences between energy intake estimated by phone and in-person 24HR, consistent with our findings [[8], [9], [10],^12^]. In the present analysis, the degree of underreporting was smaller for both recall methods than the results from Tran et al. [7], which is likely due to collection of the WFR on day prior to the 24HR. Moreover, the participants in our study consumed a relatively homogeneous diet and commonly serve food using the same portion size estimation tools employed in the survey.

The CCCs between phone recalls and WFR were higher than those comparing in-person recalls and WFR, except for calcium, riboflavin, folate, and vitamin B-12. Although there are no defined cutoffs for assessing the sufficiency of CCCs in dietary assessment, researchers have suggested that 0.40 to 0.59 indicates moderate agreement, while values between 0.60 and 0.79 provide strong evidence of agreement [[41], [42], [43]]. The CCCs were >0.60 for energy, protein, and eight micronutrients assessed by phone recall, and for seven micronutrients assessed by in-person recall. Moreover, the CCCs between phone recalls and WFR were >0.50 for energy and all nutrients, whereas only two nutrients (fat and vitamin B-6) fell <0.50 in in-person recalls. Bland-Altman plots illustrated that phone recalls had slightly less bias for protein, fat, and all but one micronutrient (vitamin B-12) than those estimated by in-person recall. Neither method had a clear advantage with respect to variability in the agreement between nutrient intake estimates, regardless of the magnitude of intake.

To our knowledge, only 2 studies have compared micronutrient intakes estimated by phone-based 24HR to those from observed or interviewer-administered WFR. Kirkpatrick et al. [13] assessed validity for vitamin A, vitamin C, folate, iron, and calcium and found no statistically significant differences between observed and reported intakes [13]. Similarly, Dubois et al. [14] found no difference between observed and reported intakes of calcium, iron, thiamine, riboflavin, niacin, vitamin C, but a lower mean intake of vitamin A relative to observed values. Both studies conducted the WFR at controlled feeding sites and included consumption of many pre-portioned and individually packaged food items, which are not commonly consumed in our study sample. Moreover, both studies provided participants with standardized portion size estimation tools for use during the phone interview. These differences may explain the higher degree of similarity between observed and reported nutrient intakes relative to our findings.

The proportion of omissions and intrusions was low in our study, and did not appear to follow any particular patterns. A small set of foods (sugar, brewed black tea, milk powder, and coconut milk-based sauce) accounted for the majority of omissions across both recall modes. These items are typically consumed in small quantities and eaten habitually, which may have contributed to underreporting. Explicitly incorporating probes for these commonly forgotten items into multiple-pass 24HR protocols could further reduce omission error in future dietary surveys in Sri Lanka and similar settings. Although our analysis did not directly compare the mean intake of food groups between the recall methods, the differences between reported and actual consumption of sugar and milk powder were smaller for phone recalls than for in-person recalls. In populations where memory error may be a concern, pictorial recall aids provided to respondents in advance of phone interviews may minimize omissions, particularly of commonly forgotten foods like snacks and beverages [44].

We expected that physical portion size estimation aids would give in-person recalls an advantage in terms of the accuracy of nutrient intake estimations. However, our results did not confirm this. The relative accuracy of phone 24HR in our study may be partly explained by lower respondent and interviewer bias [[45], [46], [47], [48], [49], [50]]. The increased anonymity of phone-based recalls may have yielded responses that more accurately reflect actual consumption [[48], [49], [50]]. In-person recalls may be even less accurate in populations with a higher propensity for social desirability bias [46]. We also expect that the extensive development of the portion size descriptions in previously collected surveys improved the accuracy of portion size estimates in the present study.

Finally, we compared the total and unit costs of phone-based and in-person 24HRs. Under the likely implementation scenario, phone-based interviews were approximately $68 (27%) less expensive per respondent than in-person interviews. Fixed costs, such as administration, planning, and tool development, were comparable across methods and largely independent of sample size. In contrast, the cost of in-person data collection was more than three times that of phone-based data collection, assuming no transportation and accommodation expenses are incurred by enumerators for phone-based interviews.

Fieldwork-related expenses for in-person surveys, including transportation, meals, and lodging, scale directly with the number of enumerators and respondents, whereas the marginal cost per additional respondent in phone-based surveys is considerably lower, reflecting minimal variable costs beyond enumerator time and phone airtime. This suggests that although both methods may benefit from economies of scale, the relative cost advantage of phone-based surveys would likely widen in larger surveys. Moreover, because this analysis was conducted in the context of a validation study, start-up costs were distributed across a smaller sample, likely inflating the unit cost. Nevertheless, unit costs for both methods were in the lower range of those reported in the literature, with the main differences reflecting that costs related to the preparation of dietary reference data were not included in this study [18]. Future work should further examine how fixed and variable cost components influence scalability and cost-efficiency across contexts.

There were several limitations in our study that must be noted. Since participants volunteered to participate, they may have been more motivated to provide accurate data. Furthermore, the sample is not population-representative, and all participants were asked to stay home on the WFR days, both of which restrict the generalizability of our findings. We also acknowledge that the WFR data collection on the day prior to the 24HR interview likely improved the accuracy of the 24HR data, as participants had heightened awareness of their food intake relative to “real-world” conditions. Additionally, trimming the 24HR data to the period during which the WFR enumerator was present could have led to an underestimation of nutrient intake relative to true intakes. However, our primary objective was to assess the relative validity of phone compared with in-person 24HR, rather than the accuracy of each method compared to WFR.

This study also has several key strengths. Our study provides important evidence from a lower-middle-income country to support the use of phone-based 24HR in future dietary assessment studies. To our knowledge, all of the published literature that compares nutrient intakes estimated by phone to those reported in-person are from North America or Western Europe. In addition, in the 2019 review of studies that validated self-reported energy intake against doubly-labeled water energy expenditure estimates, only 6 of 59 studies were performed in a low or middle-income country, all of which were in Brazil [40].

Other strengths include the crossover study design, which allowed participants to serve as their own comparison group; the availability of a locally tailored food list, comprehensive standard recipe database, and country-specific food composition data; extensive enumerator training; the assessment of recall and measurement error; and the ability to capture comprehensive food records, since participants were asked to stay home on the day of each WFR. Finally, we used complementary agreement metrics to address distinct aspects of validity, with mean differences used to evaluate the accuracy of absolute intake estimates relative to WFR, and concordance metrics used to assess consistency between recall- and WFR-derived intakes across individuals.

In conclusion, our study aimed to assess the validity and extent of error in phone 24HR relative to those conducted in-person, using WFR as a reference for true intake, as well as the costs of each recall method. Phone recalls performed as well as in-person recalls across several metrics. Although memory error was slightly lower in in-person recalls, mean portion size errors were similar, and nutrient intakes from the phone recalls more closely aligned with WFR values than those reported in-person. Unit costs were lower for the phone survey than for the in-person surveys, with scenario-based analyses suggesting 27% lower costs for phone-based recalls. The results suggest that collecting 24HR interviews by telephone may be a feasible method of dietary data collection for future population-based studies and has advantages for reducing logistical and budgetary constraints. Further research is warranted to evaluate methods to improve the accuracy of portion size estimation over the phone, in addition to validation of phone-based 24HR in other contexts.

## Author contributions

The authors’ responsibilities were as follows: AG, CAJ, CPS, RS, SYH, and DKO designed the research study and oversaw all study activities. BLC and CAJ developed the dietary data collection forms, with input from CPS, RS, and HS. AM and AG created the costing protocol and tools and performed the costing analysis with input from RS and CAJ. RS, HS, TR, TP, and CAJ conducted the research. CAJ performed the statistical analyses with input from CA, CPS, and AG. The report was written by CAJ, with input from all coauthors. CAJ had primary responsibility for the final content in the manuscript and all authors: read and approved the final manuscript.

## Data availability

Data described in the manuscript, code book, and analytic code will be made available upon request.

## Funding

This study was conducted as part of the Consultative Group on International Agricultural Research (CGIAR) Resilient Cities Initiative with support from the CGIAR Initiative on Fruit and Vegetables for Sustainable Healthy Diets (FRESH). We would like to thank all funders who supported this research through their contributions to the CGIAR Trust Fund: https://www.cgiar.org/funders/. Additional salary support was provided to BLC by USDA-ARS 2032-10700-002-00D.

## Declaration of generative AI and AI-assisted technologies in the writing process

The authors used ChatGPT (OpenAI, GPT-5.2) to support language editing, sentence restructuring, and clarity of expression during manuscript revision. After using this tool, the authors reviewed and edited the content as needed and take full responsibility for the content of the publication.

## Conflict of interest

The authors report no conflicts of interest.
