# Supplementary material for: Relative Validity of Interviewer-Administered 24-Hour Recalls Collected By Telephone and In-person Compared With Weighed Food Records Among Rural Sri Lankan Adults
Source: Curr Dev Nutr. 2026 Mar 12;10(4):107672. doi: 10.1016/j.cdnut.2026.107672 (PMC13091110; doi:10.1016/j.cdnut.2026.107672)
Supplement: multimedia component 1 [file mmc1.docx]

**Supplemental Table 1. Assumptions and time allocations for cost analysis in the phone 24-hour recall evaluation study among rural Sri Lankan adults (N=103)**

| **Item** | **Sub-item** | **Parameter value** | | | **Source** |  |
| --- | --- | --- | --- | --- | --- | --- |
| ***Per design cost parameters and assumptions*** | | | | | |  |
| Admin and planning | Duration (days) | 1 | | | Expenditure records |  |
| Training | Duration (days) | 9 | | | Expenditure records |  |
| Mean interview time (mins) | In-person (mins) | 32.01 | | | Survey |  |
|  | Phone (mins) | 29.04 | | | Survey |  |
|  | Enumerator preparation time (% of total interview) | 10% | | | KII^1^ |  |
| Data collection | Actual FTE^1^ of survey interviews as recorded by enumerators | |  | | Survey |  |
|  | Ratio FTE in-person/total interview time | 0.52 | | | Survey |  |
|  | Ratio FTE phone/total interview time | 0.48 | | | Survey |  |
|  | Number of interviews in-person | 106 | | | Survey |  |
|  | Number of interviews phone | 100 | | | Survey |  |
|  | Duration (days) | 30 | | | Expenditure records |  |
| ***Time allocations****^2^* | | |  | | |  |
| Training | | FTE allocation based on agenda items | Ratio 2:1:1 for  WFR^1^:in-person:phone | | | Training agenda |
| Data collection | | FTE allocation based on expert input from PI^1^ | Ratio 2:1:1 for  WFR:in-person:phone | | | KII |
| ***Exchange rate*** | 1 USD | LKR 360 | | | Expenditure records |  |
| ***Scenario assumptions*** | |  | | |  |  |
| *Phone survey scenario 1^2^* – *same as per design expenditures, except for:* | |  | | |  |  |
| Field work data collection costs | Phone-incurred pilot and training costs, but no accommodation or field travel costs | | | | KII |  |
|  | Interview time FTE estimated using allocation estimate by PI | | |  | KII |  |
| *Phone survey scenario 2^2^* – *same as phone recall scenario 1, except for:* | |  | | |  |  |
| Data collection | Enumerator preparation time (% of total interview) | 10% | | | KII^1^ |  |
|  | Actual FTE^1^ of survey interviews as recorded by enumerators | | | | Survey |  |

^1^FTE, full-time equivalents; KII, key informant interviews; PI, principal investigator; WFR, weighed food records

^2^Per design expenditures included all costs incurred to conduct the dietary assessment study and analyze the data, excluding those associated with external research and preparation of the reference data (e.g., food lists, portion size conversions, recipe data). Relative to the per design expenditures, phone survey scenario 1 (the most likely implementation scenario) excludes the cost of enumerators’ meals, transportation, and accommodation in the field, and it applies an allocation of interview times based on estimates from the principal investigator. Phone survey scenario 2 also excludes the cost of enumerators’ meals, transportation, and accommodation in the field but applied the actual allocation of interview times based on survey findings as in the per design scenario.

**Supplemental Table 2. Equivalence testing of phone and in-person 24-hour recall (24HR) nutrient intake estimates vs. corresponding weighed food record (WFR) values, and the relative differences between the two recall methods in the phone 24HR evaluation study among rural Sri Lankan adults**^1^

|  | Reported:actual intake ratio (90% CI^2^) | | | | |
| --- | --- | --- | --- | --- | --- |
|  | **Phone 24HR  vs. WFR** |  | **In-person 24HR  vs. WFR** |  | **WFR-Phone 24HR vs. WFR-In-person 24HR** |
| Energy (kcal) | 0.87 (0.83, 0.91) |  | 0.86 (0.81, 0.91) |  | 1.01 (0.95, 1.07)^3^ |
| Carbohydrates (g) | 0.87 (0.83, 0.92) |  | 0.88 (0.84, 0.93) |  | 0.98 (0.93, 1.04)^3^ |
| Fat (g) | 0.83 (0.77, 0.89) |  | 0.80 (0.74, 0.87) |  | 1.04 (0.95, 1.14)^3^ |
| Protein (g) | 0.92 (0.86, 0.97)^3^ |  | 0.85 (0.80, 0.90) |  | 1.08 (1.00, 1.16) |
| Calcium (mg) | 0.95 (0.86, 1.05)^3^ |  | 0.80 (0.73, 0.88) |  | 1.20 (1.06, 1.36) |
| Iron (mg) | 0.91 (0.86, 0.97)^3^ |  | 0.82 (0.77, 0.88) |  | 1.11 (1.03, 1.19) |
| Zinc (mg) | 0.91 (0.86, 0.96)^3^ |  | 0.87 (0.81, 0.92) |  | 1.05 (0.97, 1.12)^3^ |
| Vitamin A RE (µg) | 0.90 (0.79, 1.02) |  | 0.73 (0.63, 0.85) |  | 1.26 (1.07, 1.49) |
| Thiamine (mg) | 0.90 (0.84, 0.96) |  | 0.81 (0.76, 0.87) |  | 1.11 (1.02, 1.21) |
| Riboflavin (mg) | 0.96 (0.90, 1.03)^3^ |  | 0.87 (0.81, 0.93) |  | 1.12 (1.03, 1.22) |
| Niacin (mg) | 0.90 (0.85, 0.95)^3^ |  | 0.85 (0.80, 0.91) |  | 1.05 (0.98, 1.14)^3^ |
| Vitamin B6 (mg) | 0.93 (0.85, 1.02) |  | 0.90 (0.81, 0.99) |  | 1.04 (0.91, 1.19) |
| Folate (µg) | 0.94 (0.87, 1.02)^3^ |  | 0.82 (0.76, 0.88) |  | 1.15 (1.05, 1.27) |
| Vitamin B12 (µg) | 1.01 (0.87, 1.18) |  | 0.93 (0.81, 1.06) |  | 1.12 (0.93, 1.34) |
| Vitamin C (mg) | 0.93 (0.82, 1.06) |  | 0.77 (0.66, 0.90) |  | 1.25 (1.07, 1.47) |

^1^Linear regression models included interviewer and sex as fixed effects

^2^CI, confidence interval

^3^Denotes significance, i.e., values in which the 90% confidence interval fell within the equivalence margin of 0.85-1.15

**Supplemental Table 3. Concordance correlation coefficients comparing nutrient intakes reported in phone and in-person 24-hour recalls (24HR) to their corresponding weighed food records (WFR) in the phone 24HR evaluation study among rural Sri Lankan adults (N=103)**

|  | Concordance correlation coefficient (95% CI^1^) | | |
| --- | --- | --- | --- |
|  | Phone 24-HR vs. WFR |  | In-person 24HR vs. WFR |
| Energy (kcal) | 0.60 (0.48, 0.72) |  | 0.51 (0.38, 0.64) |
| Carbohydrates (g) | 0.54 (0.41, 0.67) |  | 0.53 (0.40, 0.66) |
| Fat (g) | 0.59 (0.48, 0.71) |  | 0.46 (0.33, 0.60) |
| Protein (g) | 0.65 (0.53, 0.76) |  | 0.51 (0.38, 0.64) |
| Calcium (mg) | 0.61 (0.49, 0.72) |  | 0.66 (0.56, 0.77) |
| Iron (mg) | 0.69 (0.59, 0.79) |  | 0.60 (0.48, 0.71) |
| Zinc (mg) | 0.63 (0.52, 0.75) |  | 0.50 (0.37, 0.63) |
| Vitamin A RE (µg) | 0.81 (0.74, 0.87) |  | 0.71 (0.61, 0.80) |
| Thiamine (mg) | 0.67 (0.56, 0.77) |  | 0.62 (0.50, 0.73) |
| Riboflavin (mg) | 0.66 (0.55, 0.77) |  | 0.68 (0.58, 0.77) |
| Niacin (mg) | 0.68 (0.58, 0.78) |  | 0.61 (0.50, 0.72) |
| Vitamin B6 (mg) | 0.53 (0.40, 0.66) |  | 0.46 (0.33, 0.59) |
| Folate (µg) | 0.52 (0.38, 0.66) |  | 0.57 (0.46, 0.69) |
| Vitamin B12 (µg) | 0.52 (0.38, 0.66) |  | 0.66 (0.55, 0.77) |
| Vitamin C (mg) | 0.62 (0.50, 0.74) |  | 0.60 (0.49, 0.71) |

^1^CI, confidence interval
