# Supplementary material for: Relative Validity of Interviewer-Administered 24-Hour Recalls Collected By Telephone and In-person Compared With Weighed Food Records Among Rural Sri Lankan Adults
Source: Curr Dev Nutr. 2026 Mar 12;10(4):107672. doi: 10.1016/j.cdnut.2026.107672 (PMC13091110; doi:10.1016/j.cdnut.2026.107672)
Supplement: multimedia component 2 [file mmc2.docx]

**Supplemental Figure 1. Bland-Altman plots illustrating the differences in nutrient intakes between phone and in-person 24-hour recalls (24HR) and their corresponding weighed food records (WFR) in the phone 24HR evaluation study among rural Sri Lankan adults (N=103)**^1^


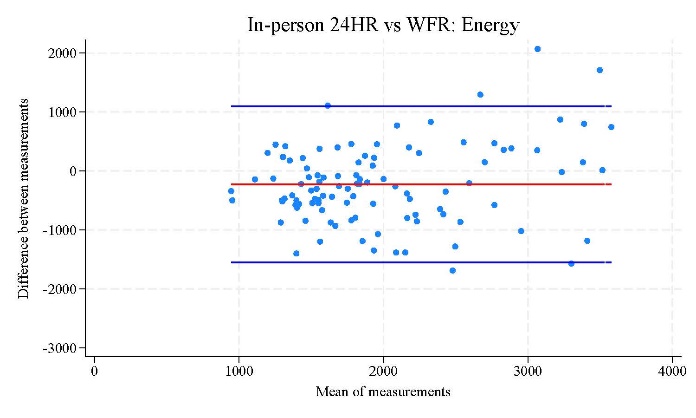

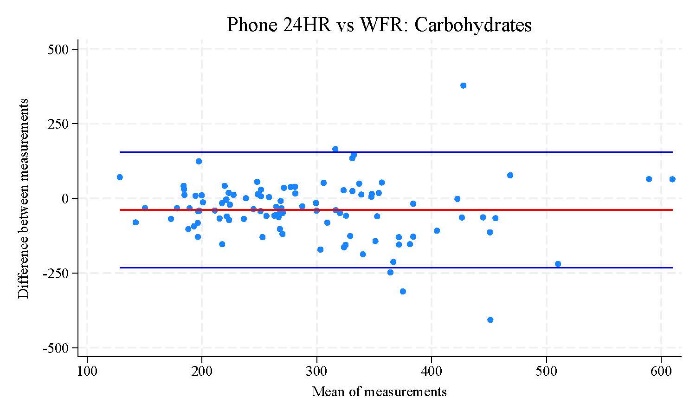

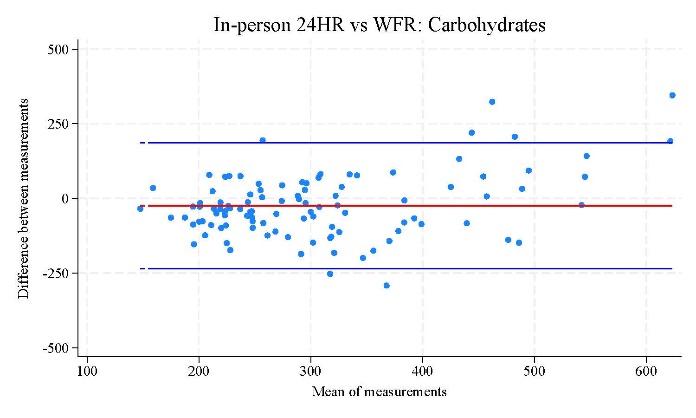

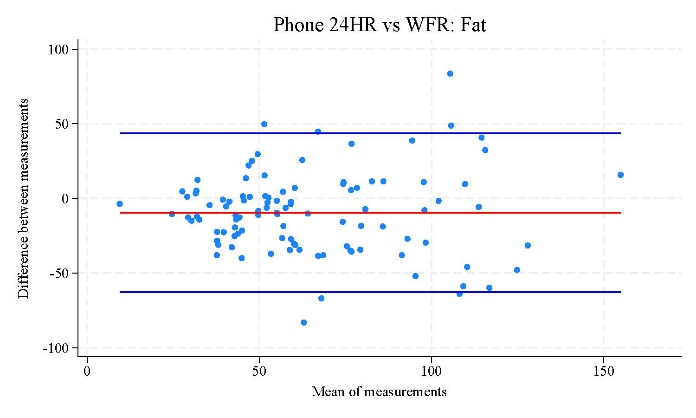

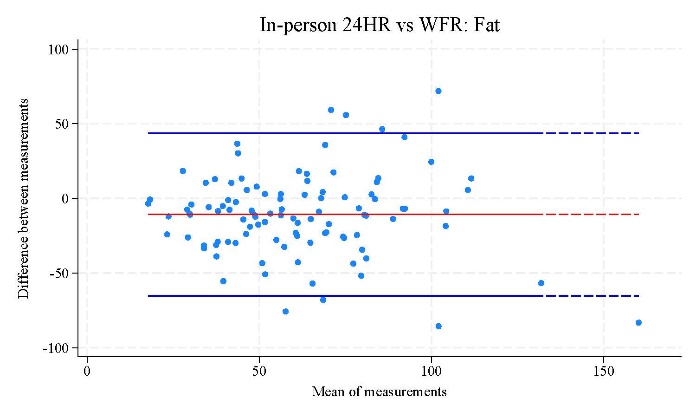

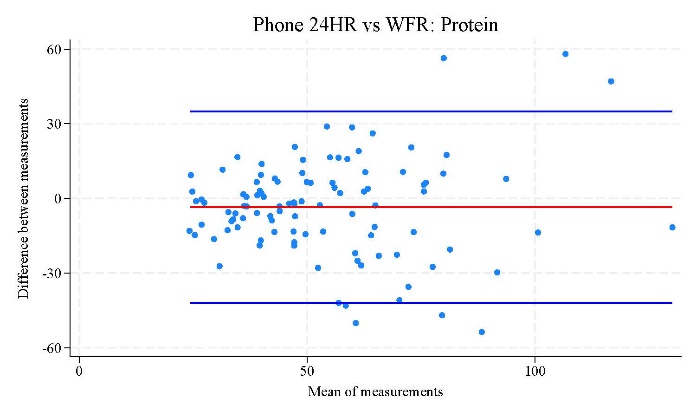

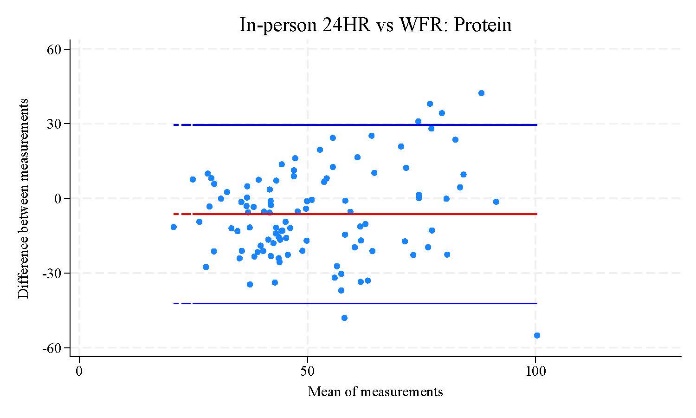

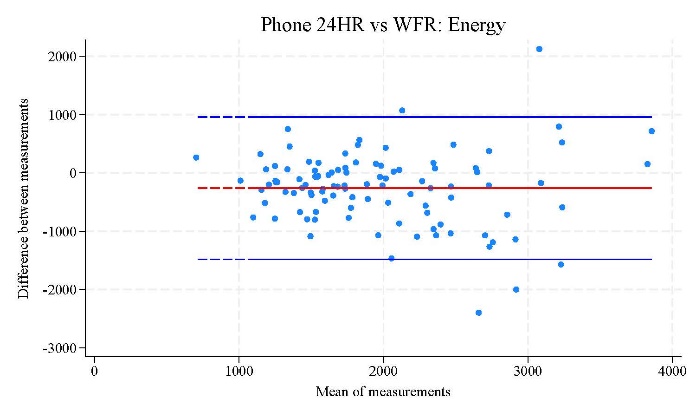

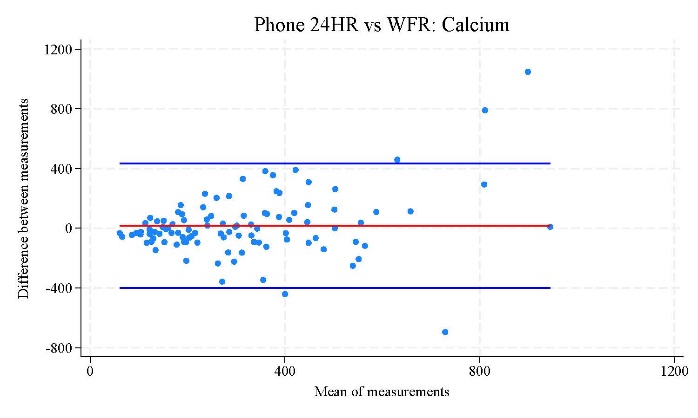

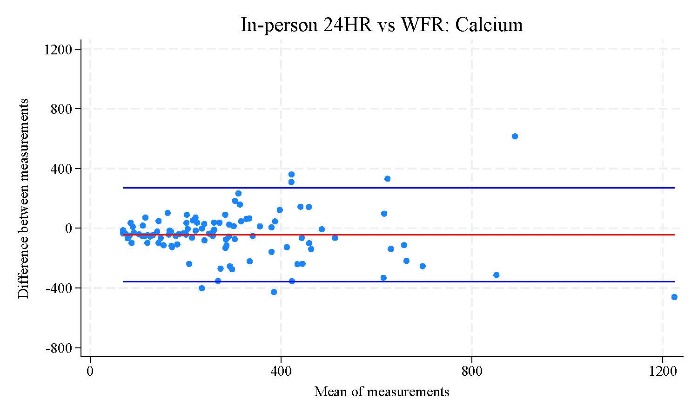

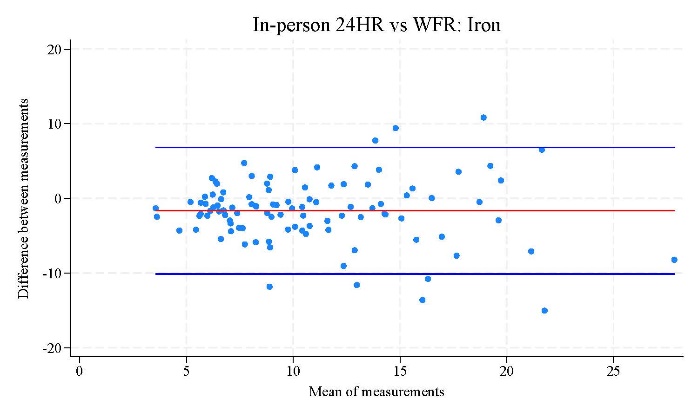

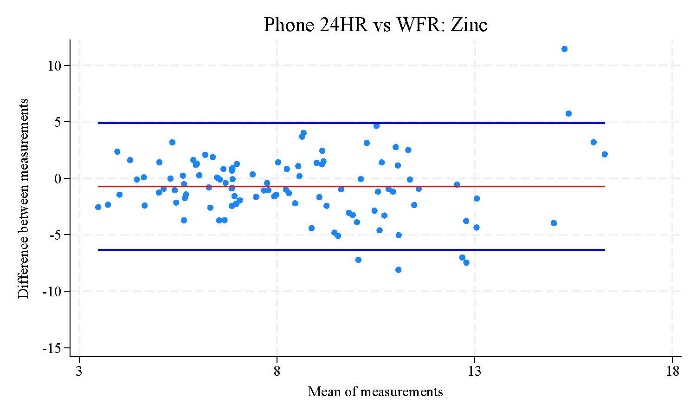

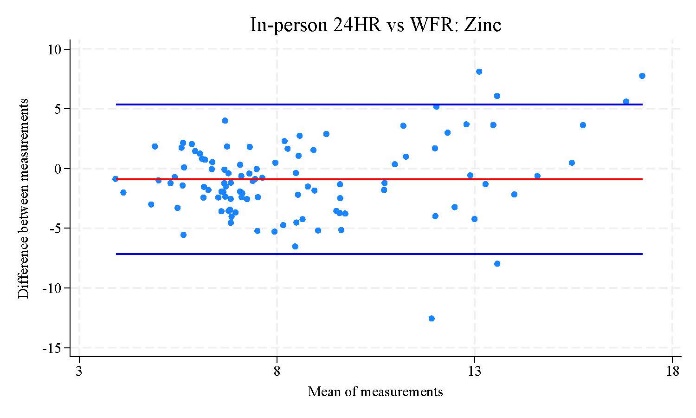

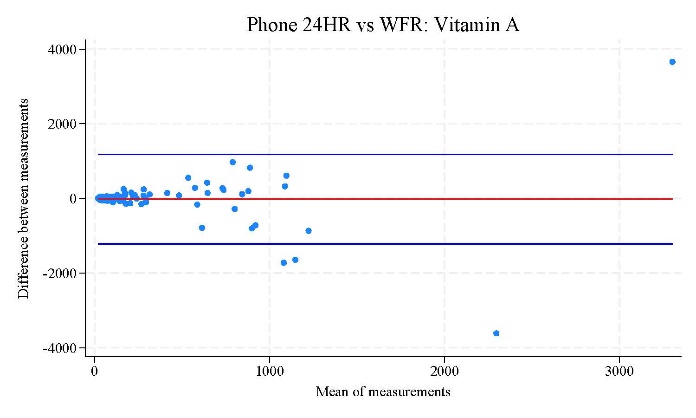

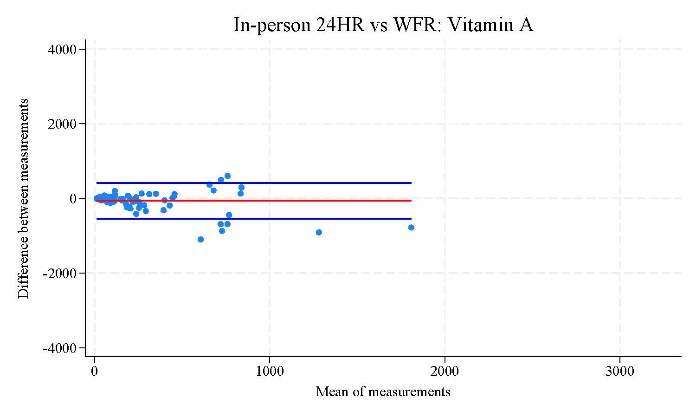

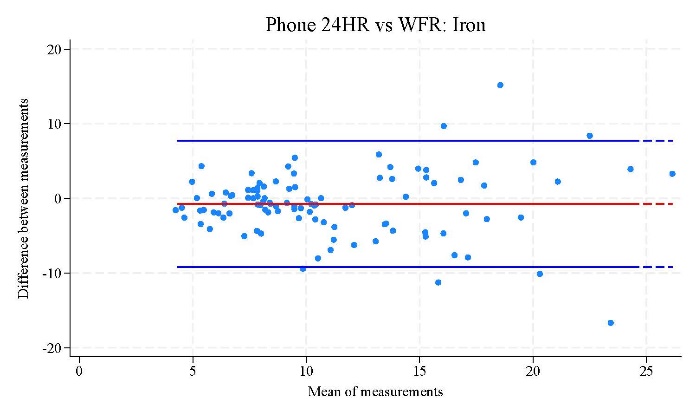

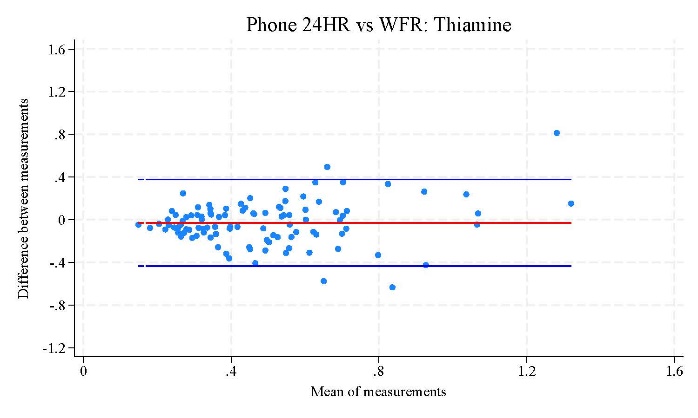

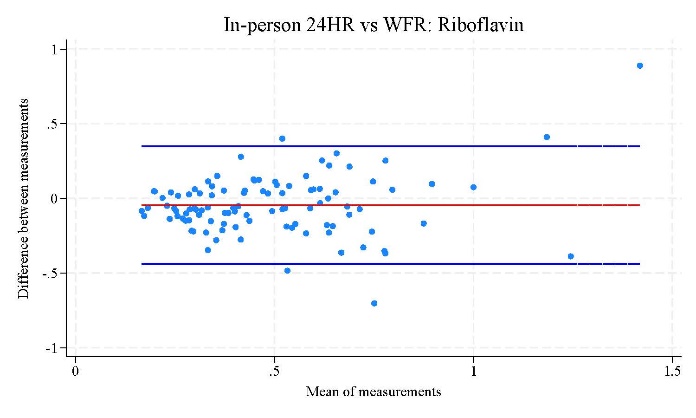

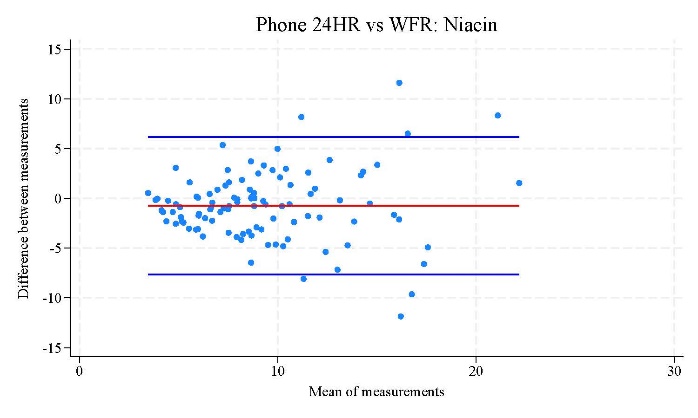

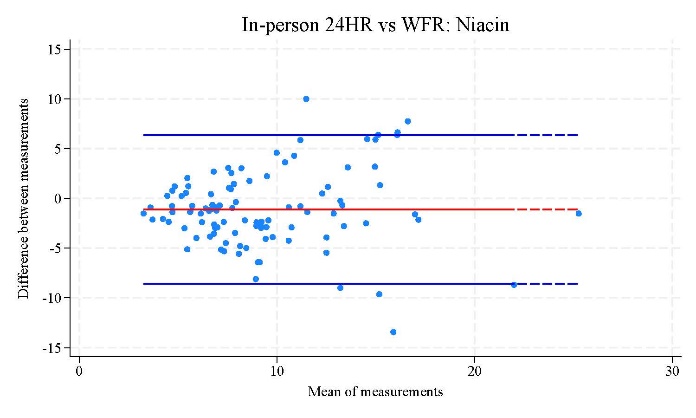

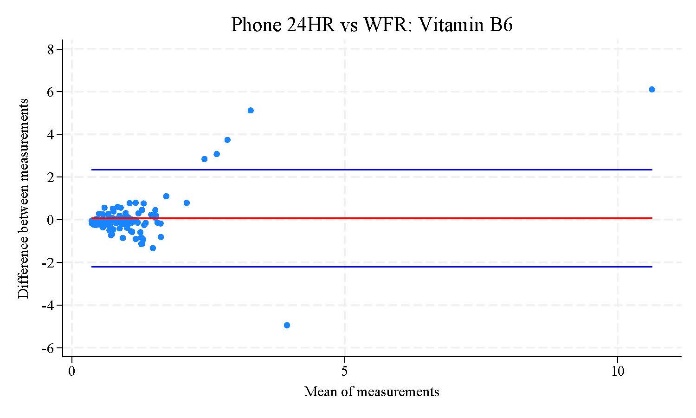

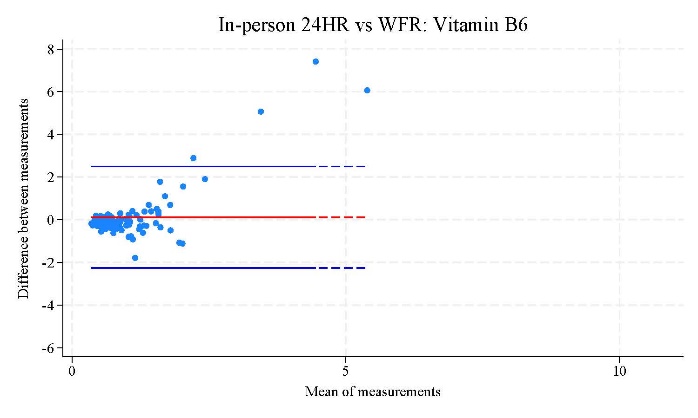

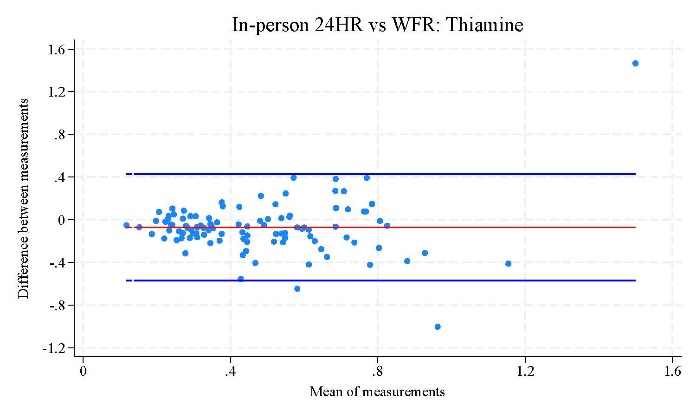

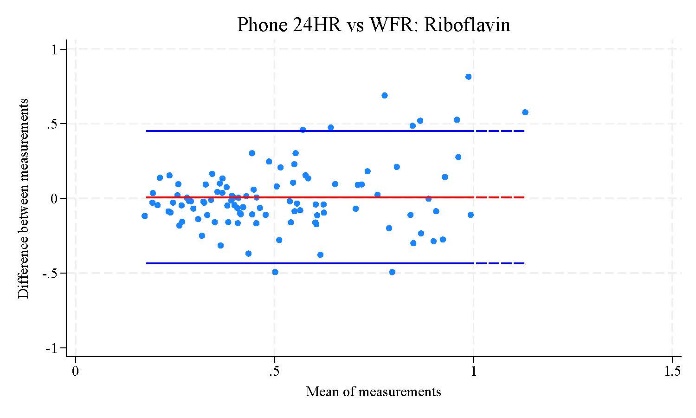

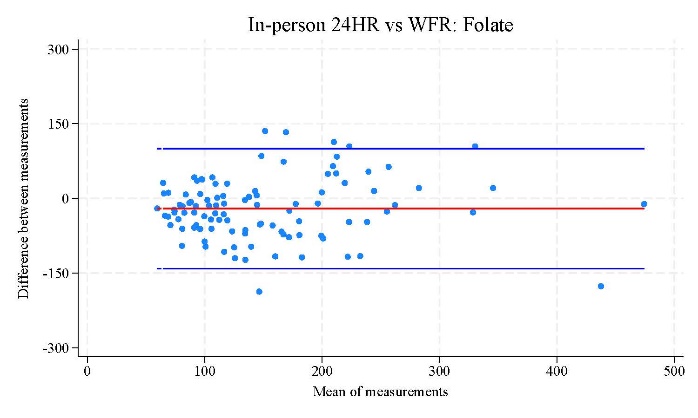

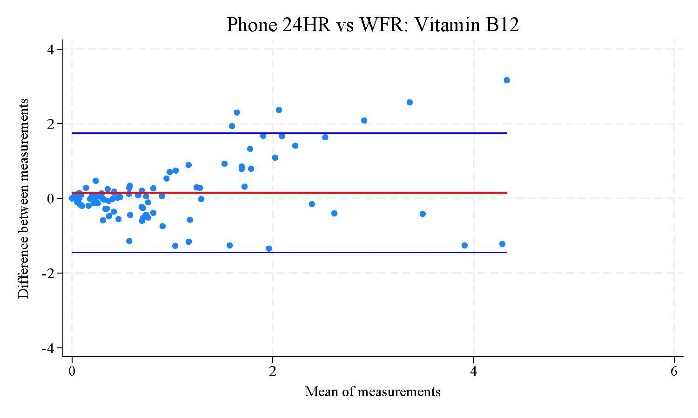

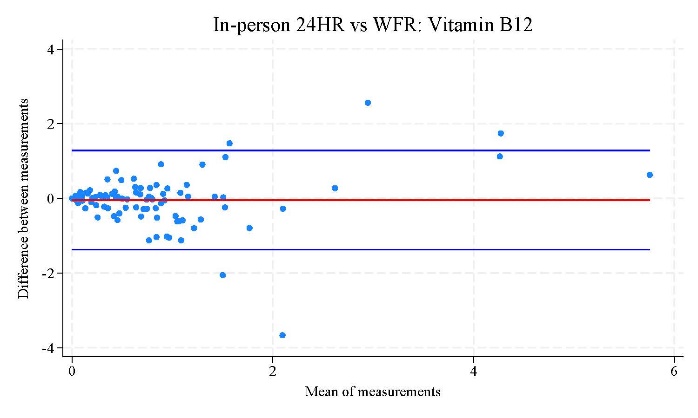

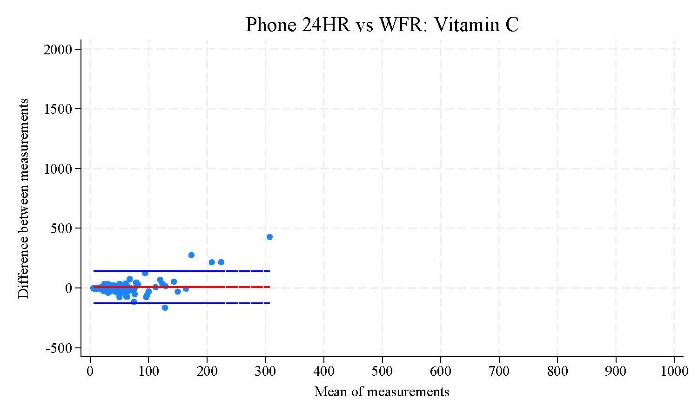

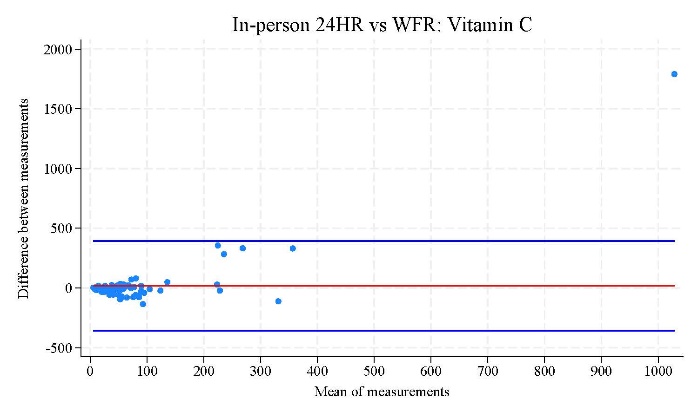

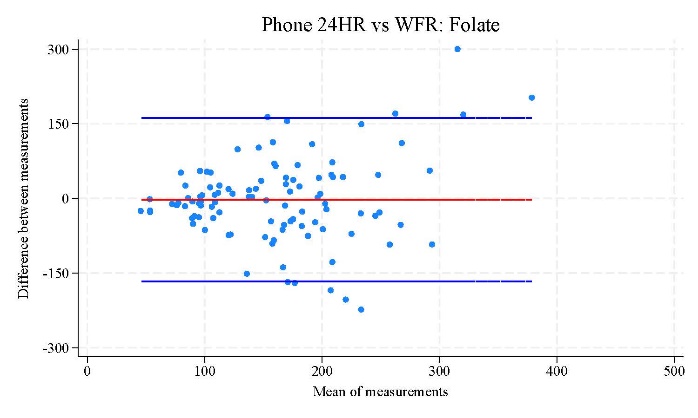


^1^The red horizontal line is the mean difference in nutrient intakes between in-person and phone 24-hour recalls (24HR) and their corresponding weighed food records (WFR), expressed as a ratio since the data was log-transformed. The blue horizontal lines are the upper and lower limits of agreement (mean +/- 1.96 SD) containing 95% of the values.
