# Supplementary material for: Relative Validity of Interviewer-Administered 24-Hour Recalls Collected By Telephone and In-person Compared With Weighed Food Records Among Rural Sri Lankan Adults
Source: Curr Dev Nutr. 2026 Mar 12;10(4):107672. doi: 10.1016/j.cdnut.2026.107672 (PMC13091110; doi:10.1016/j.cdnut.2026.107672)
Supplement: multimedia component 3 [file mmc3.docx]

**Supplemental Figure 2. Estimated unit costs for dietary assessment by cost category in the phone 24-hour recall evaluation study among rural Sri Lankan adults (N=103)**

Per design^1^

^1^Per design expenditures included all costs incurred to conduct the dietary assessment study, excluding those associated with external research and preparation of the reference data (e.g., food lists, portion size conversions, recipe data). Relative to the per design expenditures, phone survey scenario 1 (the most likely implementation scenario) excludes the cost of enumerators’ meals, transportation, and accommodation in the field, and it applies an allocation of interview times based on estimates from the principal investigator. Phone survey scenario 2 also excludes the cost of enumerators’ meals, transportation, and accommodation in the field but applied the actual allocation of interview times based on survey findings as in the per design scenario.

^2^Procurement included equipment and incentives for respondents and field officers. Training costs included salaries and accommodation at the University during enumerator training. Survey preparation and data collection included all staff salaries, transportation, and accommodation in the field during pilot testing and the data collection period (as applicable), respectively.
